# Omalizumab and IgE in the Control of Severe Allergic Asthma

**DOI:** 10.3389/fphar.2022.839011

**Published:** 2022-03-10

**Authors:** Yasuhiro Gon, Shuichiro Maruoka, Kenji Mizumura

**Affiliations:** Department of Internal Medicine, Division of Respiratory Medicine, Nihon University School of Medicine, Tokyo, Japan

**Keywords:** asthma, IgE-targeted therapy, omalizumab therapy, CD23, free-IgE

## Abstract

Omalizumab, a human immunoglobulin (Ig)G1 antibody against IgE, is a therapeutic agent for bronchial asthma. The Global Initiative for Asthma guidelines indicate that the use of omalizumab should be considered as an option in step 5 of treatment for patients with the most severe type of bronchial asthma. In patients with atopic asthma who are at a high risk of exacerbation, and in whom symptoms are poorly controlled despite treatment with inhaled corticosteroids, omalizumab is one of the few drugs that improves symptoms, reduces the risk of exacerbation, and improves the quality of life while offering a high level of safety. On the other hand, the associated treatment costs are high, and there are no clear methods to identify responders. A recent study suggested that evaluating the therapeutic effects and monitoring the pharmacokinetics of omalizumab could improve the success of omalizumab therapy. This review outlines the relationship between IgE-targeted therapy and the serum level of IgE to enhance the current understanding of the mechanism of omalizumab therapy. It also describes the clinical significance of measuring serum free IgE levels and monitoring omalizumab therapy.

## Introduction

Immunoglobulin (IgE) was discovered by Ishisaka and his wife, Teruko, in 1968 (Ishizaka, 2016. This antibody plays a key role in type I allergic reactions by functioning as a trigger for mast cell activation. Omalizumab is a human IgG1 antibody against IgE. It was approved in the United States in 2003 and in Europe in 2005 as an antibody drug for asthma; to date, it has been approved in 46 countries. In the Global Initiative for Asthma (GINA) guidelines, omalizumab is considered for the treatment of patients with the most severe type of asthma as an add-on therapeutic agent at step 5 of treatment; add-on treatment options for patients with uncontrolled severe asthma in step 4 of treatment (moderate or high dose ICS/LABA ± recommended add-on therapies) (https://ginasthma.org/gina-reports/).

In patients with refractory atopic asthma, omalizumab is one of the few treatment methods that offers a high level of safety while improving symptoms, reducing the risk of exacerbation, and improving the quality of life. Furthermore, its use can help reduce the risk of drug-induced side effects by avoiding the use of systemic steroids (Menz et al., 1998). Although omalizumab is indicated for severe atopic asthma, the extent to which IgE contributes to asthma in individual patients remains unclear.

Clinical trials examining the efficacy of omalizumab therapy have reported that there are limitations to the current diagnostic methods for atopic asthma based on serum IgE levels and intradermal reactions (Humbert et al., 1996; Menz et al., 1998; de Weck, 2002; Chung et al., 2014). In this review, we outline current fundamental biological and immunological knowledge, as well as the pharmacokinetics of IgE, that should be understood in the context of omalizumab treatment. We also discuss the clinical significance of measuring serum free IgE levels in omalizumab therapy.

## Omalizumab Therapy Indications and Therapeutic Effects

Omalizumab treatment is considered for both adults and children aged ≥6 years with serum total IgE levels of 30–150 IU/ml and poorly controlled severe atopic asthma despite treatment at step 5, according to the GINA guidelines. The therapeutic effects of omalizumab therapy are determined at 16 weeks, at which time it is recommended to examine whether treatment should be continued. The European Respiratory Society and American Thoracic Society guidelines regarding severe asthma note that, although the increased use of medical resources is an issue, omalizumab appears to be beneficial (Busse et al., 2001).

Several clinical studies have demonstrated the effectiveness of omalizumab in the treatment of severe asthma (Ayres et al., 2004; Djukanović et al., 2004; Humbert et al., 2005; Busse et al., 2011). In a Cochrane collaboration review of 19 clinical trials, upon examining the effect of omalizumab therapy as an adjunct to oral steroids, observations from weeks 16–60 revealed a significant inhibitory effect on asthma exacerbation (odds ratio [OR]: 0.55; 95% confidence interval [CI]: 0.42–0.60). Exacerbation was observed in 26% of the placebo group, in contrast to only 16% of the omalizumab therapy group (Normansell et al., 2014). Furthermore, it was reported that, during observations from weeks 28–60, the risk of hospitalization due to exacerbation was 3% in the placebo group and 0.5% in the omalizumab therapy group, with a significantly lower risk of hospitalization in the latter group (OR: 0.16; 95% CI: 0.06–0.42) (Normansell et al., 2014).

Identifying a group of patients who respond to omalizumab therapy can help reduce the unnecessary use of medical resources. A previous study has shown that serum total IgE levels are not associated with the therapeutic effects of omalizumab (Bousquet et al., 2007). The EXTRA study was conducted to examine potential biomarkers to identify responders to omalizumab therapy. The results indicated that omalizumab therapy was highly effective in patients with serum periostin (a serum marker for Th2 inflammation) levels of ≥50 ng/ml, a peripheral blood eosinophil count of ≥260 cells/μL, and fractional inhaled nitric oxide levels ≥19 ppb (Hanania et al., 2013). However, a patient population with these characteristics does not constitute an independent phenotype, and some evidence of overlap was noted between the above conditions (Arron et al., 2013).

Based on clinical trial data, the treatment response aginst omalizumab therapy is recommended to be evaluated after 16 weeks of therapy. However, assessing response before 16 weeks may not identify 100% of patients who would respond to omalizumab (Holgate et al.,.2009).

Long-term administration of omalizumab improves asthma outcomes in real-world environments such as exacerbations, hospitalization, symptoms and QOL scores without adversely affecting the risk of side effects (Korn et al., 2009; Mansur et al., 2017; Casale et al., 2019).

Allergic asthma often coexists with comobidities, that are share a common underlying allergic inflammatory mechanism. IgE-mediated immunologic pathways present an attractive target for intervention in asthma and corbidities, including allergic rhinitis, rhinoconjunctivitis, atopic dermatitis, vernal keratoconjunctivitis, chronic rhinosinusitis with nasal polyps, food allergies, and allergic bronchopulmonary aspergillosis (Humbert et al., 2019).

Among these comorbidities, sinusitis with nasal polyps is known as a refractory factor for severe asthma, but omalizumab is endoscopically used in severe CRSwNP, which has an inadequate response to intranasal corticosteroids. The clinical and patient-reported outcomes were significantly improved and well tolerated. These comorbidities of allergic asthma may influence asthma control, its severity, and patients’ response to treatment. Therefore, the use of omalizumab in patients with severe allergic asthma associated with allergic disorders has the potential to significantly improve asthma status. Therefore, maintaining adherence to long-term administration of omalizumab is important in the management of atopic severe asthma and may improve long-term disease prognosis.

Adherence is very important when considering the clinical adverse effects of non-compliance with asthma patients, especially for patients with severe asthma. A recent report on long-term adherence to omalizumab showed good adherence in 90.7% of patients, 87.8% of patients within 2 years; 85.9% of 2–4 years was good; patients over 4 years. Shows that the adherence rate was 100% (Campisi et al., 2020).

Pre- and post-treatment efficacy indices, ACT, and worsening asthma both showed significant differences between adherent and non-adherence patients, but increased age, improved ACT score, and 14-day dosing. Timing was shown to be significantly associated with increased adherence to treatment. High adherence to omalizumab has been demonstrated in real-world settings related to better results and management of asthma (Campisi et al., 2020).

## The Relationship Between Omalizumab Therapy and Total Serum IgE Levels

IgE has a molecular weight of 190 kDa, which is low compared to that of other antibody classes. Unlike other antibodies, it is characterized by both mast cell and basophil activation (Geha et al., 2003; Gould et al., 2003). Healthy individuals have serum IgE concentrations of 50–200 ng/ml, which is extremely low compared to levels of other antibodies such as IgA, IgG, and IgM, which range from 1 to 10 mg/ml. This relationship in humans is similar to that observed in rodents (Bell et al., 1989).

**FIGURE 1:**
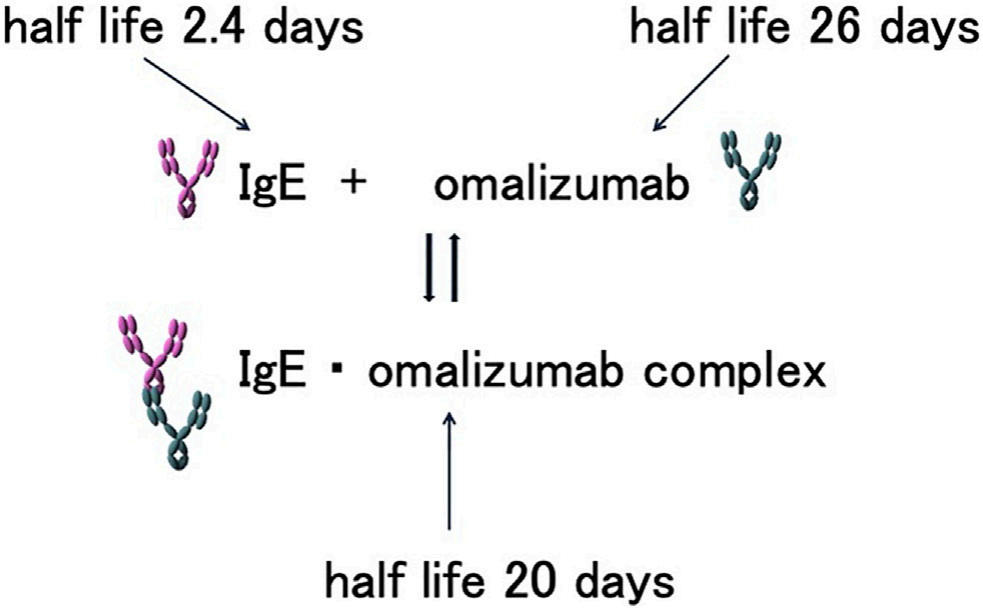
Half-lives of IgE, omalizumab, and IgE-omalizumab complexes. IgE, omalizumab, and associated complexes maintain a balanced state in the blood. The half-lives of IgE and its associated complexes differ, prolonging the half-life of serum IgE. As a result, the serum IgE concentration appears to be higher in the presence of omalizumab.

Total serum IgE levels increase after omalizumab therapy compared to pre-administration levels. This is attributed to the fact that the human serum half-life of IgE is relatively short, at 2.4 days, whereas the half-life of IgE puls omalizumab complexes is 20 days. Furthermore, omalizumab is an IgG class antibody with a long serum half-life of 26 days (Wu and Zarrin, 2014). Therefore, after binding with omalizumab, the half-life of the IgE-omalizumab complex is prolonged. As IgE simplexes and complexes cannot be distinguished using conventional total serum IgE measurement methods (e.g., ImmunoCAP Phadiotop; Thermo Fisher Scientific, Uppsala, Sweden), total serum IgE levels appear to be higher (Figure 1). The serum IgE level increases for approximately 1-2 months and then reaches a plateau (Humbert et al., 2014).

## Effects of Long-Term Omalizumab Therapy on IgE Production

Lowe and Renard (2011) presented a mathematical model based on the total serum IgE levels reported in 10 clinical trials to determine how those levels changed after several years of continuous omalizumab therapy. According to their model, total serum IgE levels gradually decreased with long-term omalizumab use. This long-term decrease also tended to increase with an increased duration of omalizumab use (Lowe and Renard, 2011). In fact, some long-term observations suggest that total serum IgE levels slightly decreased over time with long-term omalizumab therapy (Gon et al., 2018; Steiß et al., 2018).

**FIGURE 2:**
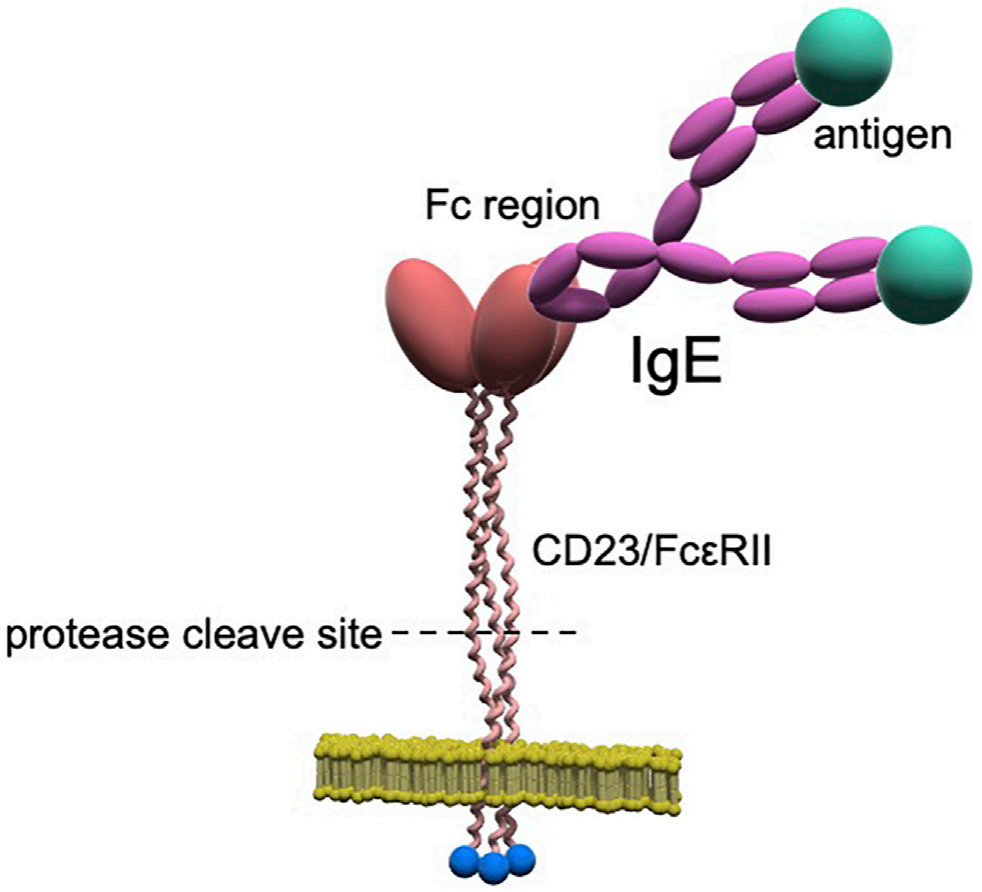
CD23/FcεRII structure. CD23 is a type II membrane protein with an intracellular N-terminus and extracellular C-terminus and forms normal trimers. The stalk portion consists of a proteolytic region cleaved by proteases (e.g., ADAMs), and the cleaved extracellular region forms soluble CD23/FcεRII.

Although the mechanism underlying the decrease in total serum IgE levels with continuous omalizumab therapy is unclear, several possibilities can be inferred. Dendritic cells (DCs) are potent antigen-presenting cells that express the high-affinity IgE receptor, FcεRI. A previous study showed that IgE is an important regulator of FcεRI expression in DCs (Poulsen and Hummelshoj, 2007). Omalizumab therapy has been thought to cause a rapid decrease in DC surface FcεRI expression and may be involved in the regulation of IgE production (Lowe and Renard, 2011). In addition, IgE production is promoted by Th2 cytokines and cluster of differentiation (CD)40 (Prussin et al., 2003); therefore, it is believed that reducing Th2 cytokine production through the inhibition of IgE could decrease IgE production. Another potential mechanism is that the reduced expression of the low-affinity IgE receptor, FcεRII (also referred to as CD23), could lead to reduced production of IgE. CD23 is a type II membrane protein with an intracellular N-terminus and extracellular C-terminus, in which the extracellular head portion binds to the Cε3 portion of the constant portion (Fc) of the IgE molecule (Figure 2) (Bacharier and Geha, 2000; Acharya et al., 2010).

High-affinity FcεRI exhibits cell-specific expression primarily on mast cells and basophils, whereas CD23 is expressed in various cells including B cells, DCs, basophils, eosinophils, epithelial cells, and smooth muscle cells. It is thought that in tissues expressing CD23, IgE binds to CD23/FcεRII, thereby forming an IgE tissue pool (Yodoi et al., 1989; Platzer et al., 2011). CD23 is also known to play a major role in the homeostasis of IgE (Platzer et al., 2011). Serum IgE is suppressed in CD23 transgenic mice, in which B cells and some T cells express high levels of CD23. This suggests that CD23 on B and T cells may cause this suppression. Moreover, in CD23 knockout mice, IgE production is increased. This is attributed to the fact that CD23 expression on B cells has an inhibitory effect on IgE production (Carlsson et al., 2007).

**FIGURE 3:**
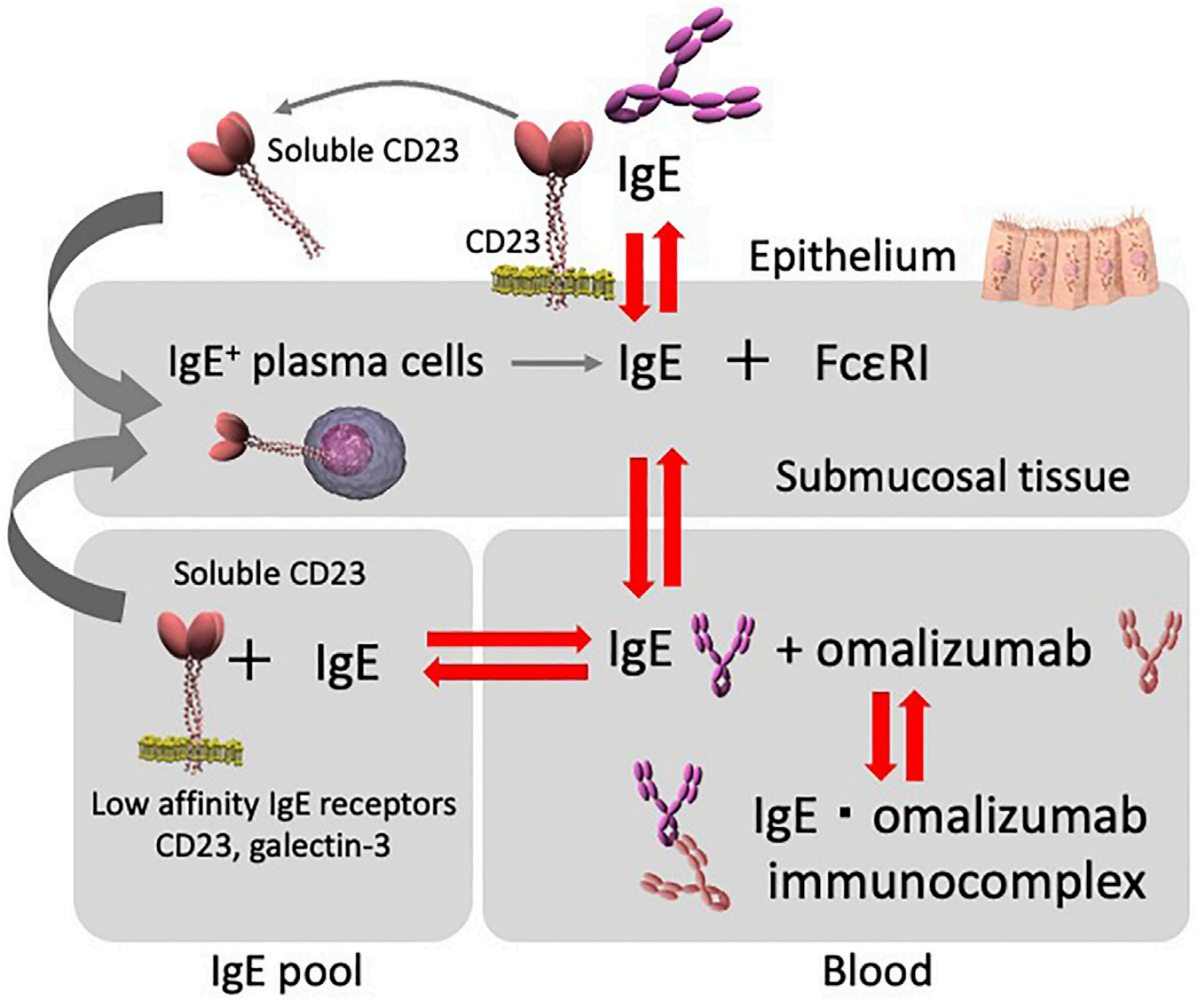
Diagram depicting the relationship between serum and tissue IgE, and omalizumab. IgE interactions. Serum IgE concentrations and the tissue IgE pool with cells expressing low-affinity IgE receptors (e.g., CD23/FcεRII) are all interconnected and regulate serum free IgE levels. Furthermore, CD23/FcεRII is involved in the inhibition of IgE production. Total serum free IgE levels during omalizumab therapy appear to be affected by each compartment.

In contrast to these findings, a soluble form of CD23 (sCD23) cleaved from membrane CD23 (mCD23) on the cell surface by proteases (e.g., ADAM17 and ADAM22) colligates both mIgE and mCD21 on the surface of IgE-producing B cells to upregulate IgE synthesis. In clinical trials of inhibitory antibodies against CD23 in humans, it was shown that the anti-human CD23 monoclonal antibody, lumiliximab, inhibits human serum IgE in a dose-dependent manner (Pène, 1989; Lamers and Yu, 1995; Poole et al., 2005). Under normal conditions, negative feedback regulation may occur when the concentration of IgE becomes high enough to allow binding to membrane CD23, thus preventing further release of soluble CD23. Although the relationship between CD23 and IgE production is interleukin-4-dependent (Reichert, 2004), it has also been reported that sCD23 is increased in allergic diseases such as asthma (Daher et al., 1995; Lorenzo et al., 1996; Richards and Katz, 1997; Rogala and Rymarczyk, 1999). Improving allergic inflammation by omalizumab therapy might reduce the overproduction of sCD23. Moreover, the binding of omalizumab to IgE consequently inhibits the binding of CD23 and IgE, thereby inhibiting mIgE and sCD23, and resulting in lower IgE production. Thus, the positive feedback loop induced by sCD23 is inhibited by omalizumab, possibly resulting in a decreased rate of IgE production (Figure 3).

As described above, the production of human IgE is basically dependent on IL-4. This can also be confirmed by the fact that IgE production is suppressed by the use of dupliumab, which is an anti-IL-4 receptor *α* antibodies. From another point of view, this means that monitoring the suppression of IgE production in the use of dupilumab may be useful in predicting the therapeutic effect. In particular, the serum IgE would be an important indicator for evaluating how dupilumab changes the atopic status of patients. In case of patients treating with dupilumab, total serum IgE is the same as free IgE, so total serum IgE is a clinical biomarker for dupilumab. However, total IgE levels may also be affected by IgE bound to the low-affinity IgE receptor CD23 (so call, IgE pool in the body). Therefore, as with free IgE when using omalizumab, it remains questionable whether total IgE sensitively reflects IgE production levels. Further consideration will be needed in the future.

## Methods of Measuring Free IgE in the Presence of Omalizumab Antibodies

Genentech’s free IgE measurement assay has been used to generate data in omalizumab clinical trials but has not been applied to general use in clinical settings (Humbert et al., 2014). Recently, several studies have evaluated the utility of commercially available assays for serum free IgE (Ohshima et al., 1995; Bencúrová et al., 2004). The antigen used in the present measurement method involves FcεRI recombinant proteins that are prepared from roundworm cells using a baculovirus vector. Thus, the glycosylation structure could differ from that found in human FcεRI, and these differences could affect its binding affinity to IgE. Baculovirus expression systems for recombinant protein production in insect cells provide immunogenic glycan structures containing *α*-1,3-core fucose (Aalberse et al., 2001), which can act as the hallmark of carbohydrate cross-reactive determinants. Such immunogenic glycan structures are often characterized by the presence of specific IgE antibodies in serum samples (Hancock et al., 2008; Seismann et al., 2010). When using the heavily glycosylated FcεRI*α* ectodomains in immunoassays, this phenomenon might evoke unexpected results.

In the IgE measurement system developed by our research group, we used antigens obtained through technology to purify human FcεRI recombinant proteins from the CHO transfectant cells expressing human FcεRI (CHO/αβγ) that secreted rsFcεRIα (Yanagihara et al., 1994; Takai et al., 2000; Takai et al., 2001). To evaluate the accuracy of our assay, we used a spike recovery assay. The recovery rates were between 80 and 120%, and the CV% values were <20% when we measured spike concentrations ranging from 9.38 ngml to 600 ngml. According to a guideline on bioanalytical method validation for clinical research by Food and Drug Administration (FDA) (https://www.fda.gov/media/70858/download), we concluded that the working ranges for this assay were as follows: lower limit of quantitation (LLOQ) = 9.38 ngml and upper limit of quantitation (ULOQ) = 600 ngml. On this method, we were able to measure serum free IgE level in the patients with omalizumab ((Ito, et al., 2014; Gon et al., 2018).

We were able to obtain near *in vivo* measurements of IgE and FcεRI (Steiß and Becher, 2014). Moreover, it is expected that serum free IgE in the presence of omalizumab that can bind to FcεRI *in vivo* can be accurately measured in patient serum.

**FIGURE 4:**
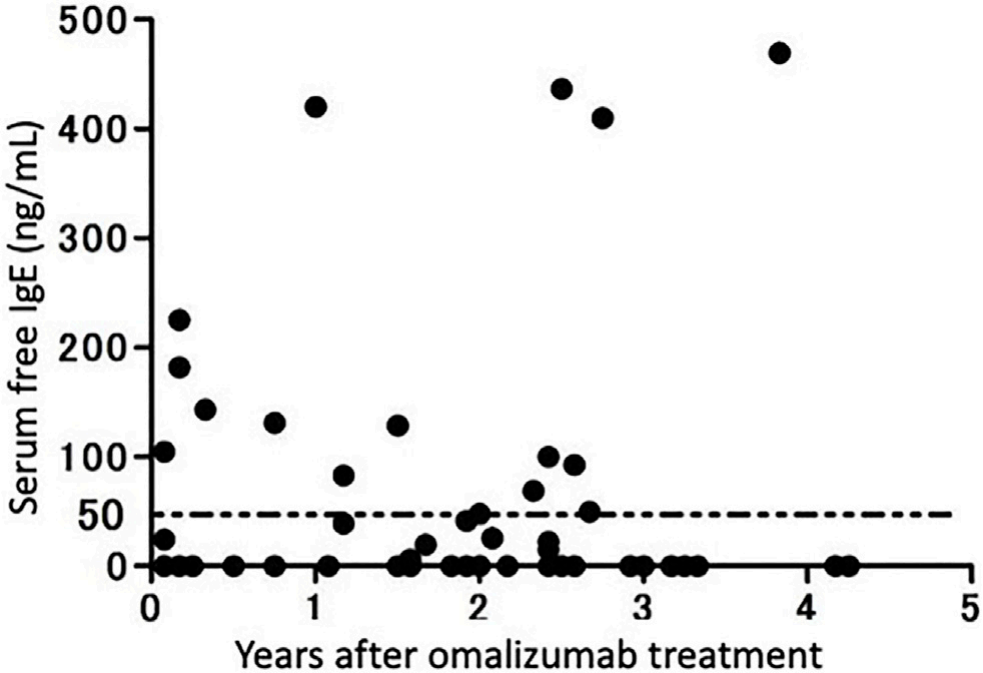
Serum IgE levels in patients receiving omalizumab therapy. Serum free IgE levels in 54 patients receiving omalizumab therapy continuously for more than 1 year were measured using an enzyme-linked immunosorbent assay (modified from Ito et al., 2014).

Using the method described above, we measured serum free IgE levels in patients who had received at least 1 month of omalizumab therapy at our institution (Figure 4) (Baker et al., 2014). Overall, 14 out of 54 patients (25.9%) met the clinical treatment target of a serum IgE level of ≥30 mg/ml. In other words, some patients, albeit a small number, did not reach the target serum IgE level in clinical practice. Therefore, measuring serum free IgE levels after a certain period of omalizumab therapy and revising the dosage is suggested as a clinical option.

Some Free IgE measurement kits are only available for research use, but there are no commercially available kits that can be used in biomarker grade for clinical settings. A free IgE measurements produced by Genentech, which have been used in many clinical studies to date, are not commercially available and cannot be used clinically, at least in Japan. The allergy center of our hospital in Japan, has accept samples from other facilities for measurements of free IgE using our method. It means that our free IgE test is the only way that is currently clinically used. One of the advantages of our measurement method that it can measure not only free IgE in the patients with omalizumab, but also total IgE in the patients without omalizumab. Since this method does not require different measurement methods for patients using omalizuamb and those not using it, the cost of the test should be reduced, and clinical application might be expected as a cost-effective test.

## Clinical Significance of Measuring Serum Free IgE Levels

Serum free IgE decreases rapidly following the administration of omalizumab, and this decrease is maintained with continuous therapy. In a clinical study of 240 patients with allergic rhinitis who were administered omalizumab for 12 weeks, although symptoms improved in the groups with serum free IgE levels of <20 ng/ml and >40 ng/ml, symptoms improved more markedly in the <20 ng/ml group. Furthermore, there was no difference observed between the <40 ng/ml, > 100 ng/ml, and <100 ng/ml groups.

Based on these results, the clinical target level of a patient’s total serum IgE in omalizumab therapy is ≤30 ng/ml (Humbert et al., 2014; Ito et al., 2014). The omalizumab dose is calculated from the patient’s pre-treatment total serum IgE levels and body weight; however, at present, there is no proposal to revise and recalculate the dose from IgE levels after initiating treatment from the study groups, and there is no specific recommendation for revising the dosage of omalizumab from the manufacturer.

Previous reports have discussed whether serum free IgE levels should be monitored during omalizumab therapy. For example, Korn et al. (2012) measured serum free IgE in 22 patients receiving omalizumab therapy and observed a mean level of 58 ± 12 ng/ml at 16 weeks. At that time, physicians identified 17 patients as responders, and there were no significant differences observed on the Asthma Control Questionnaire-5, Mini Asthma Quality of Life Questionnaire (AQLQ), or in the level of serum free IgE between the two groups. Therefore, it was concluded that the serum free IgE level was not a predictor of the therapeutic effect of omalizumab (Jardieu and Fick, 2002).

In contrast, in a prospective study utilizing our IgE measurement method and FcεRIα recombinant protein with the human glycosylation structure, the findings indicated the significance of measuring the serum free IgE level (Ito R, et al., 2014). Tajiri et al. (2016) examined the relationship between serum free IgE levels in patients with severe atopic asthma before and after 48 weeks of omalizumab therapy in relation to the asthma-related quality of life incidence of exacerbation before treatment. Patients who had experienced exacerbation 1 year before omalizumab treatment were divided into two groups, “responders”, and “incomplete responders” in this study. After the first year of omalizumab treatment, the patients who experienced exacerbations were defined as “incomplete responders,” whereas those who experienced no exacerbation in this period were defined as “responders.” The kinetics of serum free IgE levels between 16 weeks and 2 years of treatment demonstrated a differential trend between the two groups. With complete responders, mean serum free IgE levels decreased to below 30 ng/ml at 16 weeks, whereas they remained above 30 ng/ml in incomplete responders. However, after 2 years of treatment, the serum free IgE levels were below 30 ng/ml in all patient groups. In addition to the complete responders, the incomplete responders showed a significant improvement in the AQLQ and a reduced incidence of exacerbation (Tajiri et al., 2016). Furthermore, low serum free IgE levels at 32 weeks reflected a significant decrease in the incidence of exacerbation at 48 weeks. Based on these results, it was reported that measurements of serum free IgE over time in patients with severe asthma could help predict omalizumab responsiveness. We believe that these findings should be further examined in a large-scale study.

In addition to the value of using serum free IgE as a predictor of omalizumab’s therapeutic effects, it has been suggested that quantifying serum free IgE would be beneficial (Tajiri et al., 2016). Hamilton et al. (2005) noted that the measurement of serum free IgE is of clinical significance because it enables the exclusion of cases in which the omalizumab dose is insufficient, and the FcεRI-mediated activation of mast cells and basophils cannot be inhibited (Steiss et al., 2015). Furthermore, in clinical practice, when patients using omalizumab exhibit a poor response to treatment, clinicians often doubt whether patients’ serum free IgE levels are below the effective treatment range (i.e., <30 ng/ml). As IgE production is inhibited by IgE-producing B cells in an interleukin-4-dependent manner, during periods when patients are prone to allergen stimulation and upon exacerbation of the underlying pathology due to infection, IgE production is enhanced by an increase of the Th2 response, with serum IgE levels exhibiting seasonal fluctuations. In such patients, after setting the initial dosage of omalizumab, IgE production can change greatly and may surpass the serum free IgE level treatment range of <30 ng/ml. Considering the high cost of omalizumab and the fact that it is one of the few treatment options for patients with severe asthma, we believe that measuring serum free IgE levels provides considerable benefit when administering omalizumab therapy.

## Conclusion

Omalizumab is an effective molecularly targeted drug for patients with severe asthma. However, the associated healthcare costs with omalizumab are high, and there is no means to discern between responders and non-responders prior to usage. Therefore, we believe that future clinical studies that examine treatment applications of omalizumab are required. With respect to omalizumab therapy, we believe that the level of serum free IgE should be measured, and that in patients who receive long-term treatment or exhibit an insufficient response, the dosage should be reassessed by examining whether the level of serum free IgE is within the acceptable therapeutic range.
